# Effects of selenium solution on the crystalline structure, pasting and rheological properties of common buckwheat starch

**DOI:** 10.3389/fpls.2022.1053480

**Published:** 2022-12-01

**Authors:** Jiale Wang, Jiajun Leng, Licheng Gao, Mengru Han, Yixin Wu, Xinhui Lei, Jinfeng Gao

**Affiliations:** State Key Laboratory of Crop Stress Biology for Arid Areas, College of Agronomy, Northwest A&F University, Yangling, Shaanxi, China

**Keywords:** common buckwheat, selenium, physicochemical properties, starch, rheological properties

## Abstract

Selenium is an important element that affects human growth and development, and also affects the yield and quality of common buckwheat. In our study, two common buckwheat varieties were sprayed with different concentrations (0 g/hm^2^, 5 g/hm^2^, 20 g/hm^2^) of sodium selenite solution at the initial flowering period and the full flowering period, respectively, to determine the effects of selenium solution on the physicochemical properties of common buckwheat starch. With increasing selenium levels, the amylose content, peak viscosity, breakdown, relative crystallinity, pasting temperature and gelatinization enthalpy first decreased and then increased, while the transparency showed a trend of increasing and then decreasing. All samples exhibited a typical A-type pattern, while at high selenium level, the degree of short-range order of common buckwheat starches changed. From the rheological properties, it can be seen that the starch paste is dominated by elastic properties, while the low selenium treatment decreases the viscosity of the starch paste. These results showed that spraying different concentrations of selenium solutions at different periods significantly affected the physicochemical properties of common buckwheat starch.

## Introduction

Buckwheat, belongs to the *Fagopyrum* of Polygonaceae, which is native to China (Yang et al., 2010) and mainly includes two varieties: common buckwheat and Tartary buckwheat. As a food raw material, common buckwheat has great potential for the development of functional products in the food industry. Common buckwheat grains contain a variety of nutrients such as protein, starch, polysaccharides, dietary fiber, lipids, rutin, polyphenols, trace elements and macroelements (Fabjan et al., 2003; Kim et al, 2004), and its nutrient contents vary with variety and environments. In addition, the composition of amino acids in seeds of buckwheat different cultivars may vary but presence of essential amino acids is very high (Sytar et al., 2018; Sytar et al., 2018). Starch is the main form of carbohydrate storage in common buckwheat seeds with 70% of the dry weight and exists in the form of granules (Gao et al., 2020; Qin et al., 2010). Compared with wheat starch and corn starch, common buckwheat starch has higher amylose content (34%-40%), better water retention, and more stable gelatinization characteristics (Zhu, 2016)

Selenium is an essential micronutrient element and plays an important role in plant metabolism. The recommended selenium intake for humans is 55 μg/day (Navarro-Alarcon and Cdbrera-Vique, 2008), and epidemiological studies have shown that selenium intake is inversely related to mortality from various cancers (Pilon-Smits et al., 2009; Sors et al, 2005). However, low selenium intake (<50 μg/day) has become a problem for many people around the world, such as Keshan disease and Kashin-Beck disease, both caused by selenium deficiency (Navarro-Alarcon and Cdbrera-Vique, 2008). Therefore, selenium-containing extracts or selenium-enriched supplementary functional foods are more and more popular. Nowadays, the combined application of selenium in crops has become more and more extensive. Studies on pears, peaches, dates, strawberries, wheat, peanut buds and rice have shown that selenium has an impact on plants’ yield and quality (Zhang et al., 2010; Liu et al., 2020; Zapletalová et al., 2021). More and more research reports prove that selenium is also an important environmental factor affecting crop quality (Li et al., 2021), and selenium solution can improve the selenium content and starch content in crop grains (Chu et al., 2013; Kaur et al., 2018; Khalid et al., 2021). Niu et al. (2020) analyzed the contents of total selenium, organic selenium and different protein selenium in Tartary buckwheat and the forms of selenium available to the human body, indicating that the forms and compositions of organic selenium in Tartary buckwheat play an important role in human health. Humans can meet their selenium needs by eating selenium-enriched crops, so increasing the selenium content in plant products has become a current research hotspot.

The structure and physicochemical properties of crops are affected by some conditions, which are genetic background, soil and climatic conditions during their growth and development (Ahmed et al., 2019; Tappiban et al., 2020). At present, there are many studies on the physicochemical properties of buckwheat starch by nitrogen, phosphorus and other fertilizers (Zhang et al., 2019; Zhang et al., 2019), and there is a lack of research on the physicochemical properties of selenium on starch. Therefore, this experiment took two common buckwheat varieties as the main objects, and sprayed different concentrations of selenium solution (selenium-free, low selenium, high selenium) on the leaves at the initial flowering period (I) and full flowering period (F) of common buckwheat, respectively, to study the effect of selenium solution on common buckwheat starch physicochemical properties. Our aim was to reveal the effect of selenium solution on the physicochemical properties of common buckwheat starch, which was not only critical for starch-based common buckwheat products but also reduces the probability of people suffering from selenium deficiency diseases while providing food variety.

## Materials and methods

### Materials

The field experimental site was set up at the Test Station of Northwest A&F University, Yulin Academy of Agricultural Sciences, Shaanxi Province, China, located at 38°10´ north latitude and 109°46´ east longitude. This area was a semi-arid region with a continental monsoon climate in the temperate zone, with an average annual temperature of 10°C, an average annual frost-free period of 150 days, and an average annual rainfall of 400-500 mm.

Common buckwheat varieties of Xinong 9976 (XN9976) and Beizaosheng (BZS) provided by the Minor Coarse Laboratory of the Northwest A & F University were used as materials, and sodium selenite was used as selenium source, the leaves were sprayed during the initial flowering (I) and full flowering periods (F), respectively. The split zone design was adopted, and 3 selenium application levels were set to 0, 5, 20, g/hm^2^. Se0 (selenium-free), Se5 (low selenium), Se20 (high selenium) were treated with different concentrations of sodium selenite. Other practices were in conformity with local recommendations.

### Starch isolation

Mature common buckwheat seeds (500 g) were taken, pulverized using a universal high-speed pulverizer (Tianjin FW100 Co., Ltd.), and then passed through a 200-mesh sieve to obtain common buckwheat flour. Common buckwheat flour with 80% ethanol at a solid-liquid ratio of 1:20 was treated ultrasonically at 500 W and at 50°C for 30 min to remove flavonoids. A 0.3% NaOH solution (1000 mL) was added and placed at 25°C for 22 h to remove impurities such as crude fibres. This suspension was centrifuged at 4000 r/min for 10 min to remove the supernatant. After the starch was washed and precipitated with 0.3% NaOH solution, the above process was repeated thrice. Finally, the pH was adjusted to 7.0 with 0.1 mol/L HCl, and the starch was dried in an oven at 40°C and kept in a refrigerator at 4°C for later use (Zhang et al., 2019).

### Amylose content

Common buckwheat starch sample (0.01g) was weighed, placed in a 15mL centrifuge tube, 100 μl of absolute ethanol (for wetting starch) and sodium hydroxide solution (1 M, 1 mL), was added in turn, heated at 35°C for 30 minutes, added with 8.9 mL of distilled water and set as the original solution. The stock solution (200 μl was placed in a 15 mL centrifuge tube, 0.1 mol/L hydrochloric acid solution (200 μl) was added to neutralize, iodine (200 μl) was added, distilled water (9.4 mL) was added, shaken and left for 20 minutes. The absorbance was measured at 620 nm and 510 nm by using a visible-light spectrophotometer (Lab Tech Ltd., Beijing, China) (Wu et al., 2022).

### Scanning electron microscopy

The morphological characteristics of common buckwheat starch were measured by scanning electron microscope (JSM 6360 volts, All Nippon Airways, Japan). The dried starch sample was fixed on the loading platform with double-sided tape and sputtered with gold. Then the samples were observed at 20 kV and 8000x magnification.

### Granule size analysis

A laser diffraction particle size analyzer (Mastersizer 2000E, Malvern, England) was used to measure the particle size, which can measure starch samples between 0.1 and 1000 μm. Starch was added to distilled water and stirred (Zhang et al., 2019).

### X-ray diffraction analysis

XRD (D/Max 2550 VB+/PC, Riga Library, Japan Riga Library) was used to measure the crystalline structure. The powder sample was scanned in a 2θ range between 5° and 50°. At a target voltage of 40 kV, the scan rate was 10°/min (Chao et al., 2015).

### ATR-FTIR analysis

The short-range ordered structure of starch was analyzed on an FTIR spectrometer (7000, V arian, USA), which had a DTGS detector equipped with an ATR single reflection cell, and the calculated values of 1045/1022 cm^-1^ and 1022/995 cm^-1^ band strength ratio (Guo et al., 2018).

### Light transmittance

Common buckwheat starch (1 g) was accurately weighed to prepare starch homogenate with a mass concentration of 1.0%, and gelatinized in a boiling water bath for 30 min, while stirring to prevent starch from agglomerating. After the water bath, it was cooled to 25°C, distilled water was added to the original scale, and the light transmittance of the starch paste was measured with a spectrophotometer at a wavelength of 620 nm.

### Thermal properties

The thermal properties of common buckwheat starches were measured using a differential scanning calorimeter (DSC) (DSC2000, TA Instruments, USA). The dried starch sample (3.0 mg) was mixed with twice the volume of water and sealed in an aluminum pan at room temperature for 2 h. The sample pan was heated to 110°C at a rate of 10°C/min, and an empty aluminum pan was used as a control. The starch gelatinization parameters shown in the differential scanning calorimetry curve are the onset temperature (T_o_), peak temperature (T_p_), endset temperature (T_c_) and gelatinization enthalpy (ΔH) (Uarrota et al., 2013).

### Pasting properties

The pasting properties of common buckwheat starch were determined by a rapid viscosity analyzer (RVA4500, Perten, Stockholm, Sweden). The samples (2.5 g) with a moisture content of 14.0% were directly weighed into an aluminum RVA tank, and then 25.0 mL of distilled water was added to bring the total weight to 28.0 g. Set the following parameters: hold the slurry at 50°C for 1 min, heat from 50°C to 95°C for 3.7 min, and hold at 95°C for 2.5 min. Finally, let the sample cool to 50°C for 3.8 min, and keep it at 50°C for 2 min (Zhu et al., 2010).

### Rheological properties

The rheological properties of common buckwheat starch were measured by a DHR-1 rheometer from Waters Corporation, USA. The parallel metal plate of rheometer diameter was set at 40 mm and the gap at 1000 µm. The sample starch (0.4 g) and 5 mL distilled water were taken into a 10 mL centrifuge tube, and gelatinize in a boiling water bath for 10 min. The mixture was loaded onto the rheometer plate, and the gap edge was coated with a thin layer of low-density silicone oil (dimethylpolysiloxane; viscosity 50 cPa) and modified (Kong et al., 2010).

### Statistical analysis

Each experiment was repeated three times, and all data were expressed as the mean standard deviation. Data processing and graphs were analyzed using SPSS 19.0 (SPSS Inc., Chicago, Illinois, USA) and Origin software (version 2021, Microcal Inc., Northampton, Massachusetts, USA).

## Results and discussion

### Amylose content

**Figure 1:**
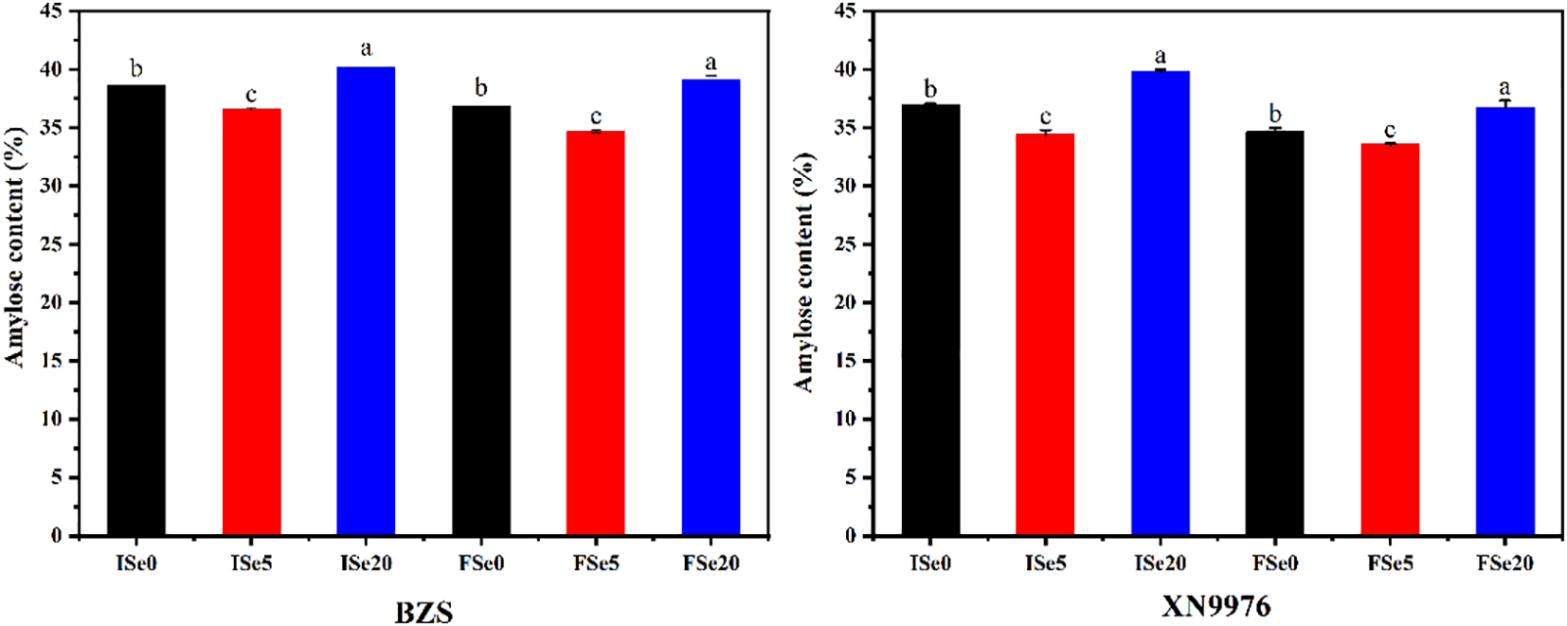
The effects of different selenium treatment levels on the amylose content of common buckwheat.

The amylose content of starch ranged from 33.58% to 40.18% and varied significantly among the different treatment levels (**Figure 1**). With increasing selenium levels, the amylose contents showed a trend of first decreasing and then increasing. The amylose contents ranged from 33.58 to 39.78% (BZS) and from 34.69 to 40.18% (XN9976), respectively. The two varieties (BZS and XN9976) had the lowest amylose content under the 5 g/hm^2^ selenium (Se5) treatment level at the two periods (initial flowering period and full flowering period), while the amylose content under the 20 g/hm^2^ selenium (Se20) treatment level was highest. Previous studies reported that the activities of soluble starch synthase (SSS) and starch branching enzymes (SBE) involved in the biosynthesis of branched components in starch granules may affect the amylose content of different starch components (Kossman and Lloyd, 2010). Kaur et al. (2018) showed that selenium treatment increased the activities of alpha, beta and total amylase, invertase and sucrose synthase, and altered the activities of enzymes related to sucrose and starch metabolism. Yuan et al. (2022) showed that excessive selenium would lead to selenium poisoning. In our study, the changes in amylose content of common buckwheat starch may be due to the altered activities of SSS, SBE and enzymes related to starch metabolism with the increase of Se levels. As for the different varieties, the amylose contents of XN9976 were higher than those of BZS, which may be related to the different genotypes (Zhang et al., 2018). Compared with spraying sodium selenite at the initial flowering period, the amylose content of sodium selenite spraying at the full flowering period did not change significantly.

Amylose content is an important indicator for evaluating starch properties. High amylose starches are characterized by high gelling strength, which can be used to produce coatings for pasta, confectionery, bread and fried products, while low amylose starches form less retrograded starch in hydrothermal product processing while having better dough kneading characteristics, especially important for noodle making (Gao et al., 2016; Gao et al., 2019). Amylose content plays a key role in the digestion of starch, as starches that are low in amylose are easier to digest than those that are high in amylose (Riley et al., 2004). Therefore, the digestion effect of common buckwheat starch treated with Se5 is better.

### Scanning electron microscopy

**Figure 2:**
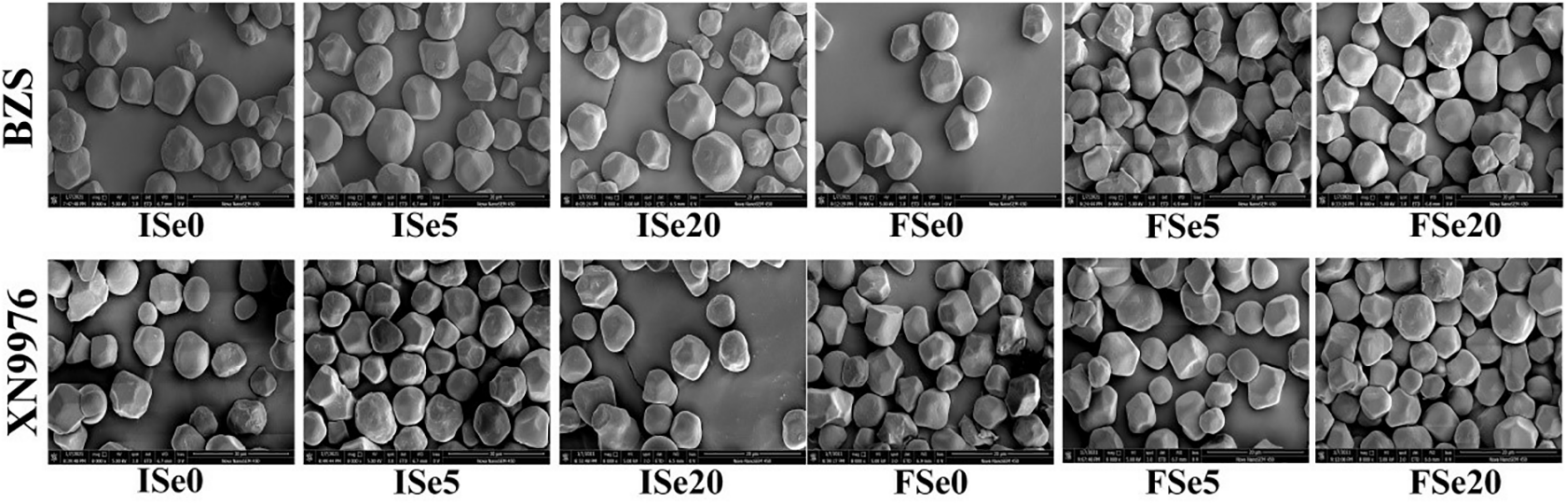
The effects of different selenium treatment levels on the morphology of common buckwheat starch granules.

The appearance and morphology of common buckwheat starch treated with different selenium were observed by scanning electron microscope (SEM) (**Figure 2**). Through observation, it could be seen that common buckwheat starches were mostly irregular polygonal particles with large volume, with obvious edges and corners. The surface of the samples had some micropores and several small spherical particles, which are similar to those of Tartary buckwheat starch (Zhang et al., 2019). Although the starch granule properties did not change after Se treatment, it was observed at different treatment levels that starch granules were relatively more minor at the Se5 treatment level.

### X-ray diffraction analysis

**Figure 3:**
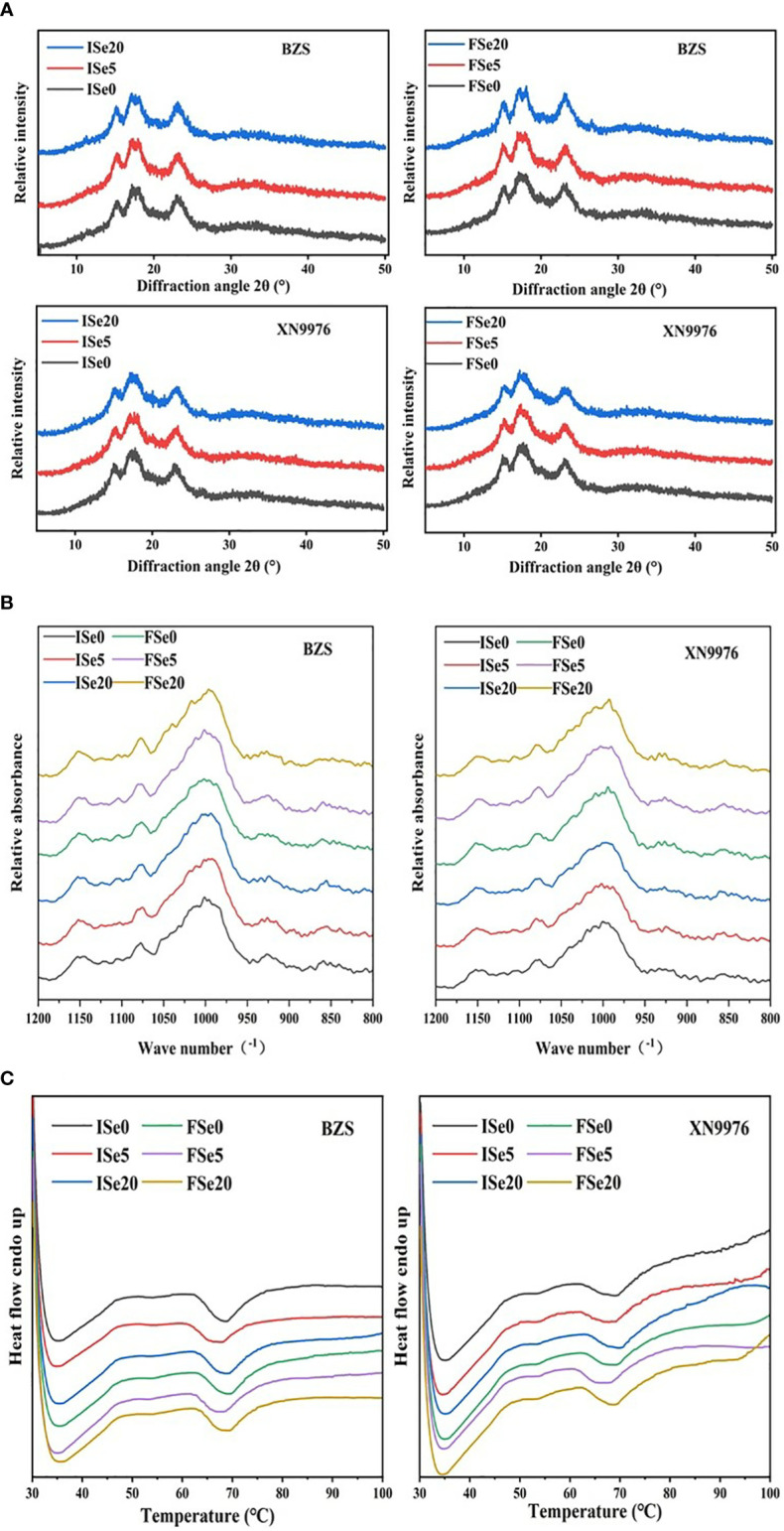
**(A–C)** X-ray diffraction patterns, Fourier peak spectrum and DSC thermogram of common buckwheat starch under different selenium treatment levels.

The XRD patterns of common buckwheat starch observed under different selenium treatment levels are shown in **Figure 3A**). Generally speaking, natural starch can be divided into A type, B type and C type according to its XRD patterns (Cheetham and Tao, 1998). All the common buckwheat starches peaked at 15°, 17°, 18°, 23° 2θ, and their X-ray diffraction patterns were typical “A” patterns. Although the X-ray diffraction patterns of common buckwheat starch did not change under different selenium treatment levels, the relative crystallinity of common buckwheat starch changed significantly (**Table 1**). With increasing selenium levels, the relative crystallinity first decreased and then increased, reaching the lowest value at the Se5 selenium level. The relative crystallinity ranged from 25.87 to 28.81% (BZS) and from 25.02 to 27.08% (XN9976), respectively. Compared with XN9976, the relative crystallinity of BZS was slightly higher. The relative crystallinity can reflect the stability of starch crystal. These results can also explain the lower gelatinization enthalpies and pasting temperature at the Se5 treatment level (**Tables 2**, **3**). Previous studies have shown that the relative crystallinity of rice starch was significantly negatively correlated with amylose content (Zhu et al., 2017), while the trend of common buckwheat starch was the opposite, and the results were consistent with the study by Gao et al. (2022). The differences may be due to genotype, cultivation management and starch extraction methods.

**Table 1:** Transparency, relative crystallinity and granule size distribution of common buckwheat under different selenium treatment level.

Varieties	Period	Treatments(g/hm^2^)	Transparency (%)	Relative	Granule size distribution (%)		
				crystallinity (%)	<5 μm	5-15 μm	>15 μm
BZS	Initial	Se 0	5.60 ± 0.06a	27.28 ± 0.29b	3.89 ± 0.13b	77.80 ± 0.24b	18.31 ± 0.07b
	flowering	Se 5	5.77 ± 0.03a	26.58 ± 0.43c	2.28 ± 0.23c	83.53 ± 0.10a	14.19 ± 0.11c
		Se 20	5.55 ± 0.04b	28.81 ± 0.31a	4.06 ± 0.05a	75.89 ± 0.00c	20.05 ± 0.05a
	Full flowering	Se 0	5.42 ± 0.05b	27.34 ± 0.43b	3.52 ± 0.02b	79.60 ± 0.17b	16.88 ± 0.08b
		Se 5	5.62 ± 0.04a	25.87 ± 0.06c	2.67 ± 0.04c	81.93 ± 0.00a	15.40 ± 0.10c
		Se 20	5.45 ± 0.06a	27.98 ± 0.10a	3.69 ± 0.03a	74.70 ± 0.08c	21.61 ± 0.11a
XN9976	Initial	Se 0	5.60 ± 0.07a	26.88 ± 0.04b	4.79 ± 0.10b	77.02 ± 0.24c	18.19 ± 0.12a
	flowering	Se 5	5.69 ± 0.11a	25.47 ± 0.21c	3.68 ± 0.11c	82.46 ± 0.05a	13.86 ± 0.14c
		Se 20	4.87 ± 0.12b	27.08 ± 0.20a	5.51 ± 0.04a	79.04 ± 0.21b	15.45 ± 0.08b
	Full flowering	Se 0	5.77 ± 0.06a	26.03 ± 0.19b	4.05 ± 0.11a	79.06 ± 0.15c	16.89 ± 0.14a
		Se 5	5.86 ± 0.11a	25.02 ± 0.12c	3.81 ± 0.09b	82.43 ± 0.08a	13.76 ± 0.08b
		Se 20	5.45 ± 0.05b	26.99 ± 0.25a	3.08 ± 0.05c	79.93 ± 0.28b	16.99 ± 0.15a

Different letters within a column indicate significant difference among mean values (p < 0.05).

**Table 2:** Thermal properties and FT-IR ratios of common buckwheat starch under different selenium treatment levels.

Varieties	Period	Treatments	Thermal properties				FT-IR ratios	
			T_o_ (°C)	T_p_ (°C)	T_c_ (°C)	ΔH(J/g)	1045/1022(cm^-1^)	1022/995 (cm^-1^)
BZS	Initial	Se 0	63.34 ± 0.08a	69.26 ± 0.28a	80.50 ± 0.13b	10.64 ± 0.81a	0.809 ± 0.00a	0.659 ± 0.18b
	flowering	Se 5	62.07 ± 0.05b	68.16 ± 0.09b	80.53 ± 0.83b	9.14 ± 0.52b	0.807 ± 0.02a	0.676 ± 0.00b
		Se 20	63.51 ± 0.03a	69.27 ± 0.19a	81.61 ± 0.74a	10.96 ± 0.20a	0.784 ± 0.00b	0.720 ± 0.00a
	Full flowering	Se 0	63.87 ± 0.06a	69.82 ± 0.05a	80.06 ± 0.36a	10.68 ± 0.50a	0.807 ± 0.00a	0.640 ± 0.05b
		Se 5	63.31 ± 0.35b	68.75 ± 0.01b	78.73 ± 0.43b	8.96 ± 0.33b	0.804 ± 0.00a	0.685 ± 0.02a
		Se 20	63.45 ± 0.08b	69.11 ± 0.35b	78.93 ± 0.48b	10.36 ± 0.57a	0.765 ± 0.00b	0.692 ± 0.02a
XN9976	Initial	Se 0	63.72 ± 0.78b	69.98 ± 0.42b	78.58 ± 0.48a	7.55 ± 0.58b	0.824 ± 0.00a	0.689 ± 0.01a
	flowering	Se 5	63.62 ± 0.08b	69.41 ± 0.14c	77.09 ± 0.66b	6.38 ± 0.00c	0.814 ± 0.02a	0.677 ± 0.01a
		Se 20	64.33 ± 0.00a	70.50 ± 0.00a	77.48 ± 0.58b	8.46 ± 0.12a	0.812 ± 0.01a	0.654 ± 0.00b
	Full flowering	Se 0	63.42 ± 0.18a	69.51 ± 0.14a	77.19 ± 0.50b	7.71 ± 0.04b	0.831 ± 0.01a	0.665 ± 0.00b
		Se 5	62.02 ± 0.27b	68.45 ± 0.13c	77.55 ± 0.59b	6.75 ± 0.54c	0.812 ± 0.02a	0.688 ± 0.01a
		Se 20	63.27 ± 0.02a	69.23 ± 0.02a	77.92 ± 0.39a	8.82 ± 0.30a	0.824 ± 0.00a	0.656 ± 0.00b

Different letters within a column indicate significant difference among mean values (p < 0.05). T_o_: onset temperature; T_p_: peak temperature; T_c_: endset temperature; ΔH: enthalpy of gelatinization.

**Table 3:** Pasting properties of common buckwheat starch under different selenium treatment levels.

Variety	Period	Treatments	Pasting properties					
			PV (cP)	TV (cP)	BD (cP)	FV (cP)	SB (cP)	Ptem(℃)
	Initial	Se 0	3515.33 ± 43b	2439.67 ± 28b	1075.67 ± 89b	4502.00 ± 60b	2062.33 ± 59ab	73.52 ± 0.1b
	flowering	Se 5	3464.33 ± 32b	2551.33 ± 55a	913.00 ± 67c	4716.67 ± 27a	2165.33 ± 70a	72.68 ± 0.0b
BZS		Se 20	3785.00 ± 43a	2486.67 ± 19ab	1298.33 ± 50a	4483.33 ± 36b	1996.67 ± 23b	74.01 ± 0.4a
	Full flowering	Se 0	3991.33 ± 47a	2467.67 ± 51b	1523.67 ± 58a	4517.00 ± 49b	2049.33 ± 13b	74.33 ± 0.7a
		Se 5	3512.00 ± 33b	2498.67 ± 34ab	1013.33 ± 30b	4680.33 ± 62a	2181.67 ± 31a	72.78 ± 0.6b
		Se 20	3516.00 ± 63b	2475.33 ± 56ab	1054.00 ± 52b	4640.00 ± 34a	2165.67 ± 24a	74.05 ± 0.4a
XN9976	Initial	Se 0	3781.33 ± 87b	2434.67 ± 25b	1347.67 ± 37a	4634.00 ± 18b	2200.33 ± 82ab	74.28 ± 0.1ab
	flowering	Se 5	3754.00 ± 6b	2416.00 ± 10b	1338.00 ± 4a	4618.67 ± 32b	2202.67 ± 30ab	74.0 ± 0.4ab
		Se 20	3967.67 ± 75a	2594.00 ± 13a	1373.67 ± 50a	4850.67 ± 45a	2256.67 ± 75a	74.78 ± 0.5a
	Full flowering	Se 0	3941.67 ± 41a	2501.67 ± 30b	1440.00 ± 63a	4619.33 ± 40b	2117.67 ± 24b	74.53 ± 0.5a
		Se 5	3664.33 ± 51c	2426.00 ± 73b	1238.33 ± 18b	4564.33 ± 73b	2138.33 ± 12b	73.71 ± 0.9b
		Se 20	3876.00 ± 57b	2613.67 ± 80a	1262.33 ± 46b	4814.33 ± 84a	2200.67 ± 45ab	74.61 ± 0.5a

Different letters within a column indicate significant difference among mean values (p < 0.05). PV: peak viscosity; TV, trough viscosity; BD, breakdown viscosity; FV, final viscosity; SB, setback viscosity; PTM, pasting temperature.

### Granule size analysis

The particle size distribution of buckwheat starch granules under different selenium treatment levels is shown in **Table 1**. There were significant differences in particle size distribution between XN9976 and BZS at different selenium treatment levels. In general, the size of starch particles was divided into three categories, namely A (>15 μm), B (5-15 μm) and C (<5 μm) (Bechtel et al., 1993). Overall, the proportion of B-granule starch was the highest (74.70%–83.53%), followed by A-granule (13.76%–21.61%) and C-granule (2.28%–5.51%). With increasing selenium levels, the proportion of large-sized (>15 μm) and small-sized (<5 μm) starch granules first decreased and then increased, and the ratio of medium-sized starch granules (5 μm–15 μm) first increased and then decreased. Large starch granules usually develop at early stages whereas small starch granules appear at late growth stages and have a low amylose content (Renuka et al., 2012). But Sing et al. showed that the amylose content was independent of the ratio of A, B and C granules (Singh et al., 2010). However, the changes in the distribution of starch granules may be related to the activity of starch synthase. There was no significant difference between the two varieties (BZS and XN9976) sprayed with selenium solution at the initial flowering period and the full flowering period.

### ATR-FTIR analysis

The crystalline region of starch was formed by the lateral arrangement of double helix branches of amylopectin into a crystal lattice, which can usually be detected by Fourier transform infrared spectroscopy (Guo et al., 2018). According to previous reports, the band ratio of 1045/1022 cm^-1^ is associated with the degree of short-range order in starch, and that of 1022/995 cm^-1^ can be used to quantify the proportion of amorphous to ordered carbohydrate structure (Guo et al., 2018; Yong et al., 2018). **Figure 3B** and **Table 2** reflected the peak value of the Fourier transform infrared spectrum of starch and the ratio of 1045/1022 cm^-1^ and 1022/995 cm^-1^ of the Fourier transform infrared spectrum of starch, respectively. It can be seen from **Table 2** that the band ratio of 1045/1022 cm^-1^ was in the range of 0.765-0.831. Under the high selenium (Se20) treatment level, the short-range orderliness changes, showing a downward trend and the band ratio of 1022/995 cm^-1^ ranged from 0.64 to 0.72. The ratio of quantitative amorphous to ordered carbohydrate structure of BZS will increase with the increase of selenium level, while the quantitative amorphous and quantitative ratio of XN9976 will decrease with the increase of selenium level.

### Light transmittance

The light transmittance (%) of common buckwheat starch paste under different varieties, periods and selenium treatment levels is shown in **Table 1**. With increasing selenium levels, the transmittance of starch pastes of two common buckwheat varieties decreased and then increased. The light transmittance of common buckwheat starch paste was the highest under the Se5 treatment. Under the Se5 treatment, the selenium contents of the two varieties were 5.77, 5.62, 5.69 and 5.86%, respectively. The results are consistent with previous research reports that amylose content is negatively correlated with the transparency of starch paste (Richard and Karkalas, 2001). Light transmittance is an important feature of starch paste, which mainly affects the sensory of food, which may affect people’s acceptance of related starch foods. Light transmittance may be affected by many factors, such as the ratio of amylose to amylopectin, particle size and storage time (Cheetham and Tao, 1998).

### Thermal properties

The thermal properties of common buckwheat starch are shown in **Table 2** and **Figure 3C**. The oneset temperature (T_o_), peak temperature (T_p_) and endset temperature (T_c_) of all common buckwheat starch the samples were 62.02~64.33°C, 68.16~70.50°C, 77.09~81.61°C, respectively. With increasing selenium levels, the onset temperature, peak temperature, endset temperature and enthalpy of gelatinization (ΔH), decreased at first and then increased. The differences in gelatinization temperature of buckwheat starch at three selenium levels may be related to amylose content, amylopectin chain length and amylose to amylopectin ratio (Zhang et al., 2019). ΔH can reflect the difficulty of starch gelatinization (Gao et al., 2019), and the range of ΔH for all samples is 6.38-10.96 J/g. Compared with other treatments, ΔH of buckwheat starch at Se5 treatment decreased significantly, which indicated that sodium selenite could make common buckwheat starch require less calories and make it easier to gelatinize. In addition, gelatinization enthalpy is commonly used to study starch crystal structure and relative crystallinity (Zhu et al., 2017). Our results are in agreement with those obtained from XRD, which suggested that starches with low ΔH at the Se5 level had lower relative crystallinity (Przetaczek-Ronowska, 2017). It can be seen from **Table 3** that ΔH of BZS was significantly higher than that of XN9976. There was no significant difference in ΔH between spraying selenium in the initial flowering period and spraying selenium solution in the full flowering period.

### Pasting properties

The pasting properties of starch significantly varied under different selenium treatment levels (**Table 3**). With increasing selenium levels, the peak viscosity (PV), breakage viscosity (BD) and pasting temperature (Ptem) showed a trend of first decline and then rise, and the setback viscosity (SB) showed a trend of first rise and then fall. Peak viscosity was the maximum viscosity of gelatinized starch when heated in water, reflecting the water binding capacity of starch granules and the degree of expansion of starch granules (Shimelis et al., 2006). The difference in peak viscosity may be due to the changes in water absorption and swelling rate of starch granules during heating. In this study, selenium reduced the swelling rate of common buckwheat starch granules, so that the starch granules slowly absorb water, thereby reducing the starch viscosity. Breakdown can reflect the heat resistance ability, the higher the breakdown viscosity, the lower the heat resistance ability (Zhou et al., 2020), indicating that the common buckwheat starch had a higher heat resistance ability under the Se5 treatment level. The pasting temperature is the temperature at which the viscosity begins to increase during heating (Kaur et al., 2009). In our study, the Se5 treatment level exhibited lower pasting temperature and easier gelatinization. The reason for this phenomenon was that higher amylose content might delay starch swelling, thereby increasing the pasting temperature (Chung et al., 2014). Setback is an indicator to measure the stability of starch paste after cooling, and high setback viscosity indicated that starch had a retrogradation trend (Uarrota et al., 2013). Trough viscosity can reflect the shear resistance of starch at high temperatures, which is an important factor affecting the food processing operation (Jiang et al., 2020). Final viscosity is due to the reduced movement of water molecules surrounded by amylose and amylopectin, reflecting the regenerative properties of starch. In our study, the tough viscosity and final viscosity of BZS sprayed sodium selenite during the initial flowering period and the full flowering period both increased first and then decreased. The opposite was true for XN9976. The reason for this phenomenon may be due to the different genotypes between breeds. Compared with spraying sodium selenite during the initial flowering period and the full flowering period, the peak viscosity, tough viscosity and damage value at the initial flowering period were slightly lower than those in the full flowering period. Pasting properties of starch are affected by amylose and lipid contents and by branch chain-length distribution of amylopectin. Amylopectin contributes to swelling of starch granules and pasting, whereas amylose and lipids inhibit the swelling (Jane et al., 1999). The differences in gelatinization characteristics may be helpful to select the appropriate samples for a specific industrial application.

### Rheological properties

**Figure 4:**
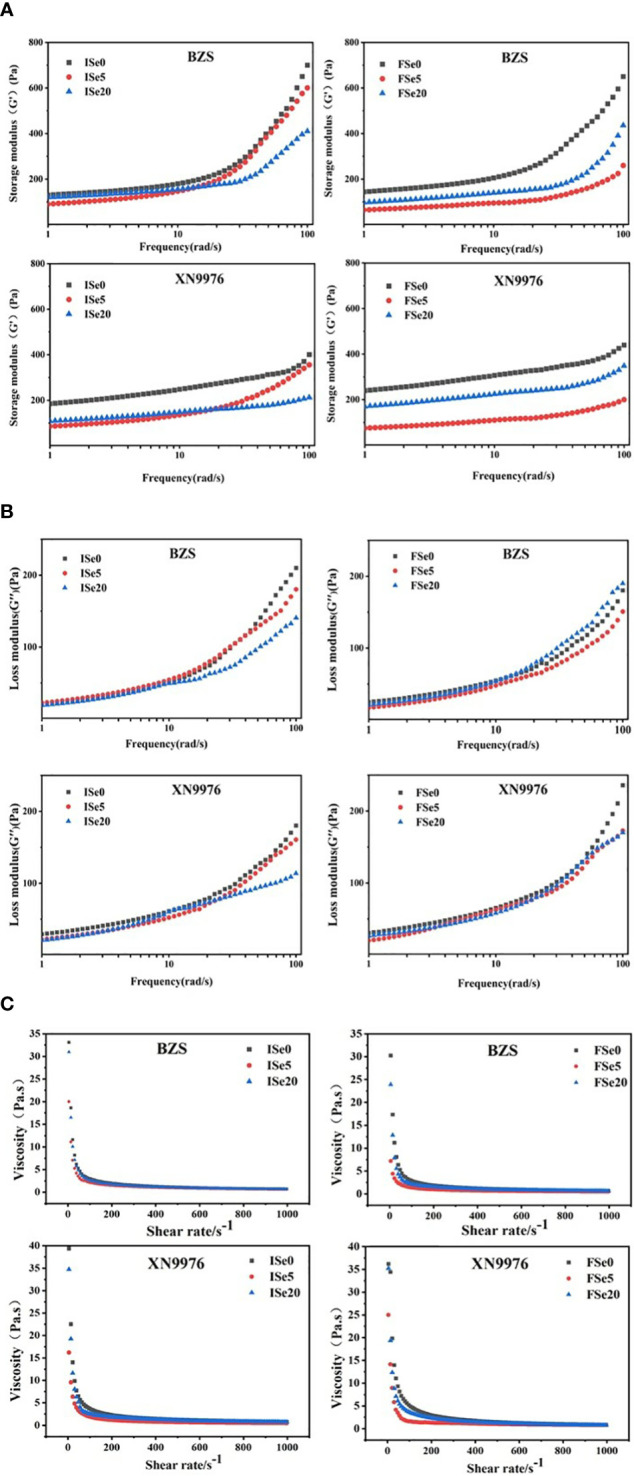
**(A, B)** Curves of storage and loss modulus of common buckwheat starch with frequency under different selenium treatment levels; **(C)** The static rheology of common buckwheat starch under different selenium treatment levels.

The food undergoes deformation after receiving the external force and has elasticity and after the force disappears, it is mainly viscous, showing a fluid state. Storage modulus (G’) and loss modulus (G’’) are used to determine the elasticity and viscosity of starch. Storage modulus (G’) was a measure of the energy stored in the material and recovered from each cycle. It was the ratio of elastic stress to strain; Loss modulus (G’’) was a measure of energy consumed or lost in each sinusoidal deformation cycle, and is the ratio of viscous stress to strain (Katopo et al., 2002). The G’ and G’’ curves of the starches are shown in **Figures 4A, B**). With the increase of frequency, the storage modulus and loss modulus of both selenium-free starch and selenium-treated starch showed an upward trend. This indicated that the viscoelasticity of the selenium-free treatment and the selenium treatment were both frequency-dependent, and the viscoelasticity increased with the increase in frequency. And under the treatment level of low selenium (Se5), the viscoelasticity of starch decreased. Gao et al. (2022) showed that lower amylose content had lower G’, which was consistent with this study. The reason may be that the addition of amylose inhibits the expansion of starch granules (Lii et al., 1996). In addition, the molecular weight and shape of amylopectin may also affect its changes. With the increase of frequency, G’ and G’’ of selenium-free starch and selenium-treated starch gradually increased, and G’ was always greater than G’’, indicating that the starch paste was mainly elastic. However, there was no significant difference in spraying sodium selenite between the two varieties, and between the initial flowering periods and full flowering periods.

The ability of starch pastes to resist fluidity is called viscosity. The size is measured by viscosity, and the viscosity of starch paste is reduced by shearing. The relationship between the viscosity of the common buckwheat starch paste treated with different selenium concentrations and the shear rate is shown in **Figure 4C**). As the shear rate increases, the viscosity drops rapidly, eventually tends to be flat, and finally approaches zero. This may be caused by the destruction of the grid structure during the shearing process, which indicated that both the selenium-treated and non-selenium-treated buckwheat starch pastes were pseudo-rapid fluids. Under the same shear rate, the viscosity of the selenium-free starch was the largest, and the viscosity of the starch decreases under the treatment level of low selenium (Se5). Under the action of shear force, starch molecules are not oriented in time, resulting in a decrease in viscosity. Xie et al. (2009) showed that higher amylose content led to higher apparent viscosity within the same shear rate range. However, there are few studies about the effects of selenium solution on the rheological properties of different cereal starch at present, so further research is needed.

## Conclusions

In our study, two buckwheat varieties were grown under three levels of selenium treatment and were treated with selenium at the initial flowering and full flowering period, respectively, and changes in the structural and physicochemical properties on common buckwheat starch affected by selenium solution were investigated. Low selenium (Se5) application significantly reduced the amylose content of common buckwheat starch, and with increasing selenium levels, peak viscosity, pasting temperature, breakdown viscosity and gelatinization enthalpy of the two varieties first decreased and then increased, while the transparency first increased and then decreased. Selenium solution changed the relative crystallinity, short-range order, storage modulus and loss modulus of starch but not its crystalline structure. In general, selenium solution changed the physicochemical properties of common buckwheat starch, and there were significant differences in the physicochemical properties of starch between the two varieties. A future goal is to explore how the selenium solution affects the fine structure and its physicochemical properties on common buckwheat starch.

## Data availability statement

The original contributions presented in the study are included in the article/Supplementary Material. Further inquiries can be directed to the corresponding author.

## Author contributions

JW: Writing original draft and Data curation. JL: Methodology and Data curation. LG: Software. MH: Resources. YW: Methodology. XL: Validation. JG: Funding acquisition and Writing - review and editing. All authors contributed to the article and approved the submitted version.

## Funding

This research was funded by the National Key R&D Program of China (2020YFD1000805-03), National Natural Science Foundation of China (31671631), Shaanxi Science and Technology Key R&D Program (2022NY-178), Shaanxi Province Minor Cereals Industrial Technology System (NYKJ-2021-YL (XN) 40).

## Conflict of interest

The authors declare that the research was conducted in the absence of any commercial or financial relationships that could be construed as a potential conflict of interest.

## Publisher’s note

All claims expressed in this article are solely those of the authors and do not necessarily represent those of their affiliated organizations, or those of the publisher, the editors and the reviewers. Any product that may be evaluated in this article, or claim that may be made by its manufacturer, is not guaranteed or endorsed by the publisher.

## References

[B1] Ahmed S. Ru W. Han H. Cheng L. Bao J. (2019). Fine molecular structure and its effects on physicochemical properties of starches in potatoes grown in two locations. Food Hydrocolloids. 97, 105172. doi: 10.1016/j.foodhyd.2019.105172

[B2] Bechtel D. B. Zayas I. Dempster R. Wilson J. D. (1993). Size-distribution of starch granules isolated from hard red winter and soft red winter wheats. Cereal Chem. 70, 238–240. doi: 10.1021/bp00020a017

[B3] Chao G. Gao J. Liu R. Wang L. Li C. Wang Y. . (2015). Starch physicochemical properties of waxy proso millet (Panicum miliaceum l.). Starch - Starke 66, 1005–1012. doi: 10.1002/star.201400018

[B4] Cheetham N. Tao L. (1998). Variation in crystalline type with amylose content in maize starch granules: an X-ray powder diffraction study. Carbohydr. Polymers 36, 277–284. doi: 10.1016/S0144-8617(98)00007-1

[B5] Chung H. Li X. Kalinga D. Lim T. Yada R. Qiang L. (2014). Physicochemical properties of dry matter and isolated starch from potatoes grown in different locations in Canada. Food Res. Int. 57, 89–94. doi: 10.1016/j.foodres.2014.01.034

[B6] Chu J. Yao X. Yue Z. Li J. Zhao J. (2013). The effects of selenium on physiological traits, grain selenium content and yield of winter wheat at different development stages. Biol. Trace Element Res. 151, 434–440. doi: 10.1007/s12011-012-9575-6 23250542

[B7] Fabjan N. Rode J. Kosir I. J. Wang Z. Kreft I. (2003). Tartary buckwheat (Fagopyrum tataricum gaertn.) as a source of dietary rutin and quercitrin. J. Agric. Food Chem. 51, 6452–6455. doi: 10.1021/jf034543e 14558761

[B8] Gao J. Kreft I. Chao G. Wang Y. Liu X. Wang L. . (2016). Tartary buckwheat (Fagopyrum tataricum gaertn.) starch, a side product in functional food production, as a potential source of retrograded starch. Food Chem. 190, 552–558. doi: 10.1016/j.foodchem.2015.05.122 26213009

[B9] Gao L. Wan C. Wang J. Wang P. Gao X. Mia E. . (2022). Relationship between nitrogen fertilizer and structural, pasting and rheological properties on common buckwheat starch. Food Chem. 389, 132664–132664. doi: 10.1016/J.FOODCHEM.2022.132664 35523074

[B10] Gao L. Wang H. Leng J. Wang P. Yang P. Gao X. Gao J. (2020). Structural, pasting and thermal properties of common buckwheat (Fagopyrum esculentum Moench) starches affected by molecular structure. International journal of biological macromolecules. 156, 120–126. doi: 10.1016/j.ijbiomac.2020.04.064 32289422

[B11] Gao L. Xia M. Li Z. Wang M. Gao J. (2019). Common buckwheat-resistant starch as a suitable raw material for food production: A structural and physicochemical investigation. Int. J. Biol. Macromolecules 145, 145–153. doi: 10.1016/j.ijbiomac.2019.12.116 31846660

[B12] Guo K. Lin L. Fan X. Zhang L. Wei C. (2018). Comparison of structural and functional properties of starches from five fruit kernels. Food Chem. 275, 75–82. doi: 10.1016/j.foodchem.2018.03.004 29622233

[B13] Jane J. ,. L. Chen Y. ,. Y. Lee L. ,. F. McPherson A. ,. E. Wong K. ,. S. Radosavljevic M. . (1999). Effects of amylopectin branch chain length and amylose content on the gelatinization and pasting properties of starch. Cereal Chem. 76 (5), 629–637. doi: 10.1094/CCHEM.1999.76.5.629

[B14] Jiang F. Du C. Guo Y. Fu J. Jiang W. Du S. (2020). Physicochemical and structural properties of starches isolated from quinoa varieties. Food Hydrocolloids 101, 105515. doi: 10.1016/j.foodhyd.2019.105515

[B15] Katopo H. Song Y. Jane J. L. (2002). Effect and mechanism of ultrahigh hydrostatic pressure on the structure and properties of starches. Carbohydr. Polymers 47, 233–244. doi: 10.1016/S0144-8617(01)00168-0

[B16] Kaur M. Sharma S. Singh D. (2018). Influence of selenium on carbohydrate accumulation in developing wheat grains. Commun. Soil Sci. Plant Anal. 49, 1650–1659. doi: 10.1080/00103624.2018.1474903

[B17] Kaur A. Singh N. Ezekiel R. Sodhi N. S. (2009). Properties of starches separated from potatoes stored under different conditions. Food Chem. 114, 1396–1404. doi: 10.1016/j.foodchem.2008.11.025

[B18] Khalid M. Imran M. Aslam M. Ashraf M. (2021). The interactive effect of selenium and farmyard manure on soil microbial activities, yield and selenium accumulation by wheat (Triticum aestivum l.) grains. J. Plant Growth Regulation 41, 2669–2677. doi: 10.1007/S00344-021-10465-5

[B19] Kim S. L. Kim S. K. Park C. H. (2004). Introduction and nutritional evaluation of buckwheat sprouts as a new vegetable. Food Res. Int. 37, 319–327. doi: 10.1016/j.foodres.2003.12.008

[B20] Kong X. Kasapis S. Bertoft E. Corke H. (2010). Rheological properties of starches from grain amaranth and their relationship to starch structure. Starch - Strke 62, 302–308. doi: 10.1002/star.200900235

[B21] Kossmann J. Lloyd J. (2010). Understanding and influencing starch biochemistry. Crit. Rev. Plant Sci. 19, 171–226. doi: 10.1080/07352680091139204 10907795

[B22] Lii C. Y. Tsai M. L. Tseng K. H. (1996). Effect of amylose content on the rheological property of rice starch. Cereal Chem. 73 (4), 415–420.

[B23] Liu X. Huang Z. Li Y. Xie W. Mo Z. (2020). Selenium-silicon (Se-Si) induced modulations in physio-biochemical responses, grain yield, quality, aroma formation and lodging in fragrant rice. Ecotoxicology Environ. Safety 196, 110525. doi: 10.1016/j.ecoenv.2020.110525 32224370

[B24] Li J. Yang W. Guo A. Qi Z. Wang J. (2021). Combined foliar and soil selenium fertilizer increased the grain yield, quality, total Se, and organic Se content in naked oats. J. Cereal Science 100, 103265. doi: 10.1016/j.jcs.2021.103265

[B25] Navarro-Alarcon M. Cdbrera-Vique C. (2008). Selenium in food and the human body: A review. Sci. Total Environment 400, 115–141. doi: 10.1016/j.scitotenv.2008.06.024 18657851

[B26] Niu Q. Dong R. Messia M. C. Ren T. Hu X. (2020). Selenium in Se-enriched tartary buckwheat (Fagopyrum tataricum l. gaertn.): Its molecular form and changes during processing. J. Cereal Science 95, 103022. doi: 10.1016/j.jcs.2020.103022

[B27] Pilon-Smits E. A. Quinn C. F. Tapken W. Malagoli M. Schiavon M. (2009). Physiological functions of beneficial elements. Curr. Opin. Plant Biol. 12, 267–274. doi: 10.1016/j.pbi.2009.04.009 19477676

[B28] Przetaczek-Ronowska I. (2017). Physicochemical properties of starches isolated from pumpkin compared with potato and corn starches. Int. J. Biol. Macromolecules 101, 536–542. doi: 10.1016/j.ijbiomac.2017.03.092 28322952

[B29] Qin P. Qiang W. Fang S. Hou Z. Ren G. (2010). Nutritional composition and flavonoids content of flour from different buckwheat cultivars. Int. J. Food Sci. Technology 45, 951–958. doi: 10.1111/j.1365-2621.2010.02231.x

[B30] Renuka N. Song X. Eric B. Koushik S. (2012). Exploring the surface morphology of developing wheat starch granules by using atomic force microscopy. Starch - Starke 65, 398–409. doi: 10.1002/star.201200172

[B31] Richard F. T. Karkalas J. (2001). The effects of environmental conditions on the structural features and physico-chemical properties of starches. Starch Strke 53, 513–519. doi: 10.1002/1521-379X(200110)53:10<513::AID-STAR513>3.0.CO;2-5

[B32] Riley C. K. Wheatley A. O. Hassan I. Ahmad M. H. Morrison E. Asemota H. N. (2004). *In vitro* digestibility of raw starches extracted from five yam (Dioscorea spp.) species grown in Jamaica. Starch Strke 56, 69–73. doi: 10.1002/star.200300195

[B33] Shimelis E. A. Meaza M. Rakshit S. (2006). Physico-chemical properties, pasting behavior and functional characteristics of flours and starches from improved bean (Phaseolus vulgaris l.) varieties grown in East Africa. Int. Commission Agric. Eng. 8.

[B34] Singh S. Singh N. Isono N. Noda T. (2010). Relationship of granule size distribution and amylopectin structure with pasting, thermal, and retrogradation properties in wheat starch. J. Agric. Food Chem. 58, 1180–1188. doi: 10.1021/jf902753f 20043631

[B35] Sors T. G. Ellis D. R. Salt D. E. (2005). Selenium uptake, translocation, assimilation and metabolic fate in plants. Photosynthesis Res. 86, 373–389. doi: 10.1007/s11120-005-5222-9 16307305

[B36] Sytar O. Biel W. Smetanska I. Brestič M. (2018a). Bioactive compounds and their biofunctional properties of different buckwheat germplasms for food processing. buckwheat germplasm in the world. Acad. Press, 191–204. doi: 10.1016/B978-0-12-811006-5.00019-7

[B37] Sytar O. Chrenková M. Ferencová J. Polačiková M. Rajský M. Brestič M. (2018b). Nutrient capacity of amino acids from buckwheat seeds and sprouts. J. Food Nutr. Res. 57 (1), 38–47.

[B38] Tappiban P. Sraphet S. Srisawad N. Wu P. Han H. Smith D. . (2020). Effects of cassava variety and growth location on starch fine structure and physicochemical properties. Food Hydrocolloids. 108, 106074. doi: 10.1016/j.foodhyd.2020.106074

[B39] Uarrota V. G. Amante R. E. Demiate M. I. Vierira F. Delgadillo I. Maraschin M. (2013). Physicochemical, thermal, and pasting properties of flours and starches of eight Brazilian maize landraces ( zea mays l.). Food Hydrocolloids. 30, 614–624. doi: 10.1016/j.foodhyd.2012.08.005

[B40] Wu W. Zhang X. Qu J. Xu R. Liu N. Zhu C. . (2022). The effects of fermentation of qu on the digestibility and structure of waxy maize starch. Front. Plant Sci 2899. doi: 10.3389/fpls.2022.984795 PMC9424902 36051290

[B41] Xie F. Yu L. Su B. Liu P. Wang J. Liu H. . (2009). Rheological properties of starches with different amylose/amylopectin ratios. J. Cereal science 49 (3), 371–377. doi: 10.1016/j.jcs.2009.01.002

[B42] Yang X. Wu Z. Chen H. Shao J. Qi W. (2010). Karyotype and genetic relationship based on RAPD markers of six wild buckwheat species (Fagopyrum spp.) from southwest of China. Genet. Resour. Crop Evolution 57, 649–656. doi: 10.1007/s10722-009-9500-9

[B43] Yong H. Wang X. Sun J. Fang Y. Liu J. Jin C. (2018). Comparison of the structural characterization and physicochemical properties of starches from seven purple sweet potato varieties cultivated in China. Int. J. Biol. Macromolecules 120, 1632–1638. doi: 10.1016/j.ijbiomac.2018.09.182 30287360

[B44] Yuan Z. Long W. Liang T. Zhu M. Zhu A. Luo X. . (2022). Effect of foliar spraying of organic and inorganic selenium fertilizers during different growth stages on selenium accumulation and speciation in rice. Plant Soil 1–15. doi: 10.1007/s11104-022-05567-2

[B45] Zapletalová A. Ducsay L. Slepan M. Vicianová M. Buo R. (2021). Selenium effect on wheat grain yield and quality applied in different growth stages. Plant Soil Environment 67, 147–153. doi: 10.17221/589/2020-PSE

[B46] Zhang H. Han T. Tian L. Wang Y. Jia H. (2010). Accumulation of Se in peach,jujube and strawberry after spraying sefertilizer on leaves. J. Fruit Science 62, 547–566. doi: 10.1103/PhysRevD.62.054027

[B47] Zhang W. Yang Q. Xia M. Bai W. Gao J. (2019). Effects of phosphate fertiliser on the physicochemical properties of tartary buckwheat (Fagopyrum tataricum (L.) gaertn.) starch. Food Chem. 307, 125543. doi: 10.1016/j.foodchem.2019.125543 31634760

[B48] Zhang W. Yang Q. Xia M. Bai W. Wang P. Gao X. . (2019). Effects of nitrogen level on the physicochemical properties of tartary buckwheat (Fagopyrum tataricum (L.) gaertn.) starch. Int. J. Biol. Macromolecules 129, 799–808. doi: 10.1016/j.ijbiomac.2019.02.018 30731161

[B49] Zhang L. Zhao L. Bian X. Guo K. Zhou L. Wei C. (2018). Characterization and comparative study of starches from seven purple sweet potatoes. Food Hydrocolloids. 80, 168–176. doi: 10.1016/j.foodhyd.2018.02.006

[B50] Zhou T. Zhou Q. Li E. Yuan L. Wang W. Zhang H. . (2020). Effects of nitrogen fertilizer on structure and physicochemical properties of 'super' rice starch. Carbohydr. Polymers 239, 116237. doi: 10.1016/j.carbpol.2020.116237 32414446

[B51] Zhu F. (2016). Buckwheat starch: Structures, properties, and applications. Trends Food Sci. Technology 49, 121–135. doi: 10.1016/j.tifs.2015.12.002

[B52] Zhu L. Liu Q. Sang Y. Gu M. Shi Y. (2010). Underlying reasons for waxy rice flours having different pasting properties. Food Chem. 120, 94–100. doi: 10.1016/j.foodchem.2009.09.076

[B53] Zhu D. Zhang H. Guo B. Xu K. Dai Q. Wei C. . (2017). Effects of nitrogen level on structure and physicochemical properties of rice starch. Food Hydrocolloids. 63, 525–532. doi: 10.1016/j.foodhyd.2016.09.042

